# Family-Based Next-Generation Sequencing Study Identifies an *IL2RG* Variant in an Infant with Primary Immunodeficiency

**DOI:** 10.1089/omi.2018.0196

**Published:** 2019-05-17

**Authors:** Aravind K. Bandari, Sunil Bhat, MV Archana, Sunita Yadavalli, Krishna Patel, Pavithra Rajagopalan, Anil K. Madugundu, Manisha Madkaikar, Kavita Reddy, Babylakshmi Muthusamy, Akhilesh Pandey

**Affiliations:** ^1^ Institute of Bioinformatics, Bangalore, India.; ^2^ Manipal Academy of Higher Education, Manipal, Karnataka, India.; ^3^ Center for Molecular Medicine, National Institute of Mental Health and Neurosciences (NIMHANS), Bangalore, India.; ^4^ Pediatric Hematology, Oncology and Bone Marrow Transplant, Mazumdar Shaw Medical Center, Narayana Health City, Bangalore, India.; ^5^ Amrita School of Biotechnology, Amrita Vishwa Vidyapeetham, Kollam, India.; ^6^ Department of Laboratory Medicine and Pathology, Mayo Clinic, Rochester, Minnesota.; ^7^ Center for Individualized Medicine, Mayo Clinic, Rochester, Minnesota.; ^8^ National Institute of Immunohaematology, KEM Hospital Campus, Mumbai, India.

**Keywords:** molecular diagnostics, bone marrow transplantation, primary immunodeficiency, genetic defects, next-generation sequencing, newborn screening

## Abstract

Primary immunodeficiencies (PIDs) are a rare and heterogeneous group of inherited genetic disorders that are characterized by an absent or impaired immune system. In this report, we describe the use of next-generation sequencing to investigate a male infant with clinical and immunological manifestations suggestive of a PID. Whole-exome sequencing of the infant along with his parents revealed a novel nucleotide variant (cytosine to adenine substitution at nucleotide position 252) in the coding region of the interleukin 2 receptor subunit gamma (*IL2RG*) gene. The mother was found to be a carrier. These findings are consistent with a diagnosis of X-linked severe combined immunodeficiency and represent the first such reported mutation in an Indian family. This mutation leads to an asparagine to lysine substitution (p.Asn84Lys) located in the extracellular domain of IL2RG, which is predicted to be pathogenic. Our study demonstrates the power of next-generation sequencing in identifying potential causative mutations to enable accurate clinical diagnosis, prenatal screening, and carrier female detection in PID patients. We believe that this approach, which is not a current routine in clinical practice, will become a mainstream component of individualized medicine in the near future.

## Introduction

Primary immunodeficiencies (PIDs) are a group of chronic genetic disorders in which a part of the immune system is missing or incompletely developed (Bousfiha et al., 2015). Delayed diagnosis of PID increases the risk of morbidity and mortality of the patients. The 2017 report of International Union of Immunological Societies (IUIS) has classified the known PIDs into nine major categories that include (1) immunodeficiencies affecting cellular and humoral immunity, (2) combined immunodeficiencies with associated or syndromic features, (3) predominantly antibody deficiencies, (4) diseases of immune dysregulation, (5) congenital defects of phagocyte number, function, or both, (6) defects in intrinsic and innate immunity, (7) autoinflammatory disorders, (8) deficiency of the complement system, and (9) phenocopies of PID (Bousfiha et al., 2018).

Among the PIDs, severe combined immunodeficiency (SCID) is a potentially fatal disorder characterized by markedly decreased or absent circulating T cells, normal/increased or decreased/absent circulating B cells, and decreased serum antibodies (immunoglobulin [Ig]) (Cirillo et al., 2015; Lee et al., 2011; Picard et al., 2015). The incidence of SCID is estimated to be 1 in 100,000 (Lindegren et al., 2004) newborns in the world and 1 in 58,000 in the U.S. population (Kwan et al., 2014). Patients with SCID are generally asymptomatic at birth but later present with persistent infections, especially with opportunistic pathogens, including *Acinetobacter*, *Pseudomonas*, *Aspergillus*, *Candida*, cytomegalovirus, and herpes zoster virus (Fischer et al., 2015). Affected infants develop these infections during their first few months of age and generally die before 1 year of their age, if untreated.

Mutations in 17 genes are currently known to cause SCID, which are involved in the T lymphocyte differentiation process. Among those genes, *IL2RG*, *JAK3*, *IL7R*, *PTPRC*, *CD3D*, *CD3E*, *CD247*, *CORO1A*, and *LAT* are reported to cause T-B+ SCID and *RAG1*, *RAG2*, *DCLRE1C*, *PRKDC*, *NHEJ1*, *LIG4*, *AK2*, and *ADA* are known to cause T-B- SCID (Picard et al., 2018). These genes are associated with an autosomal recessive mode of inheritance except for interleukin 2 receptor subunit gamma (*IL2RG*), which is the only causative gene located on the X-chromosome and thus associated with an X-linked recessive mode of inheritance. *IL2RG* mutations are estimated to account for 40–50% of the SCID patients (Chan and Puck, 2005; Kalman et al., 2004; Touzot et al., 2015).

*IL2RG* gene codes for the common gamma chain that acts as a signal transducing subunit for several interleukin receptors, including IL-2, IL-4, IL-7, IL-9, IL-15, and IL-21. It is therefore crucial for the development and function of both the innate and adaptive immune systems (Asao et al., 2001; Conley et al., 1999; Rochman et al., 2009). The IL2RG protein has a signal peptide (1–22), an extracellular domain with Ig repeats (23–262), a transmembrane domain (263–283), and a short cytoplasmic tail (284–369) (Bai et al., 2015). IL2RG is predicted to contain six potential N-linked glycosylation sites at positions 24, 71, 75, 84, 159, and 249 (UniProt ID: P31785).

The common gamma chain is crucial for IL-2 signaling as it forms an oligomeric complex with IL-2 receptor-alpha and -beta subunits in response to IL-2 stimulation and activates Janus kinases (JAK1 and JAK3), which lead to downstream activation of signal transducer and activator of transcription 5 (STAT5) (Picard et al., 2015), phosphoinositide 3-kinase signaling (PI3K), and mitogen-activated protein kinase (MAPK) pathways (Nakarai et al., 1994; Picard et al., 2015; Waickman et al., 2016).

Owing to the heterogeneous clinical presentation and high mortality rates, rapid diagnosis is important to initiate treatment regimens such as hematopoietic stem cell transplantation and enzyme replacement therapy that can significantly improve the survival of children with SCID (Ferrua and Aiuti, 2017; Kohn and Gaspar, 2017). Here, we report the clinical and immunological manifestations of a male infant who presented with repeated infections. Whole-exome sequencing of the infant and his parents revealed a novel nucleotide variant c.252C>A in the *IL2RG* gene that resulted in an amino acid change of asparagine to lysine (p.Asn84Lys) as a potential cause for the SCID. Our study highlights the importance of next-generation sequencing approach as a means to identify potential causal variants in clinically and genetically heterogeneous diseases such as SCID.

## Materials and Methods

### Ethics statement

This study was approved by the institutional ethics committee (Narayana Health Academic Ethics Committee).

### Patient history

A male infant born to nonconsanguineous parents from the southern part of India was enrolled in this study. The patient was normal at birth, but at 6 months of age presented with an eczematous rash and a history of repeated infections. Initially, he was treated for seborrheic dermatitis; however, the rash was later determined to be due to low lymphocyte counts. He was readmitted at the age of 11 months with convulsions and altered sensorium. Except for respiratory failure, no other organ failure was observed. The blood profile showed low hemoglobin 9.5 g/dL (normal range: 11.1–14.1) and low mean corpuscular hemoglobin 21.8 pg (normal range: 24.0–30.0). Platelet, neutrophil, and monocyte counts were also low.

**FIG. 1.**
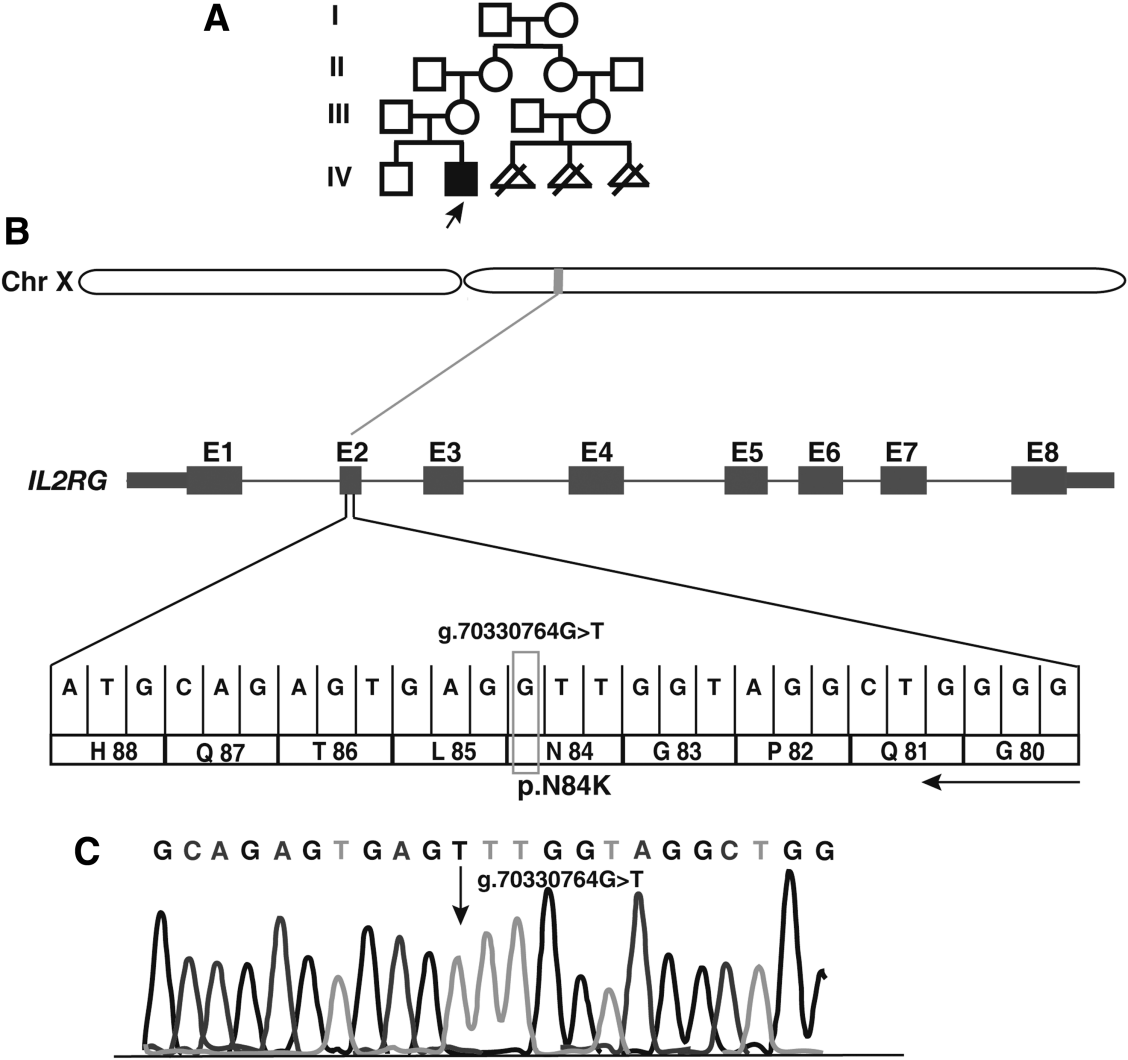
A novel mutation identified in an infant with X-linked SCID. **(A)** The family pedigree is shown where the *arrow* indicates the proband who was sequenced along with his parents. **(B)** Depiction of single-nucleotide variant g.70330764G>T identified in exon 2 of *IL2RG* gene located on the X-chromosome. The exons of *IL2RG* are shown as rectangular boxes labeled E1 through E8. **(C)** A chromatogram from Sanger sequencing-based validation of g.70330764G>T variant in the *IL2RG* gene in the patient. The mutation is indicated by the *arrow*. *IL2RG*, interleukin 2 receptor subunit gamma; SCID, severe combined immunodeficiency.

Flow cytometry tests revealed normal B cell counts with decreased T and natural killer (NK) cell counts and low levels of serum Igs (Table 1), a pattern consistent with SCID cases (Picard et al., 2015). The proband was awaiting a human leukocyte antigen-matched donor for bone marrow transplantation when he expired on developing pneumonia and sepsis at the age of 11 months. His elder brother was a healthy 3-year old and there was no history of SCID in the family. The mother's cousin had three miscarriages in early pregnancies due to unknown reasons (Fig. 1A).

**Table 1.** Flow Cytometry Findings in the Proband

*Cell type*	*Lymphocyte count (%)*	*Absolute lymphocyte count (cells/μL)*	*Reference range for absolute count (cells/μL)*
Lymphocytes	1.7	100	700–4500
CD3^+^ (T cells)	16.1	16	542–2327
CD19^+^ (B cells)	76.6	77	61–509
CD3^−^CD56^+^ (NK cells)	0.7	1	28–418
CD3^+^CD4^+^ (CD4^+^ cells)	11.9	12	350–1600
CD3^+^CD8^+^ (CD8^+^ cells)	2.6	3	300–1450
CD20^+^ (B cells)	91.2	91	66–529

NK, natural killer.

### Laboratory assessment of the patient

The patient was ascertained by trained immunologists and geneticists. Whole blood was collected from the patient and his parents after procuring informed consent from the family. Intact white blood cells were obtained from whole blood after lysis of red blood cells (RBC) with RBC lysis buffer. Leukocytes were stained with fluorochrome-conjugated anti-human cell surface markers. Cell events were captured using fluorescence-activated cell scan, and low subsets of T cells (CD3, CD4, CD8), NK cells (CD56), and low levels of serum Igs (IgG, IgA, IgM, IgE, and IgD) were observed. The number of B cells CDl9 (+) in the patient was similar to normal controls. The cell surface markers were measured using a flow cytometer and clinical, laboratory findings confirmed SCID in the patient (Table 1).

### Exome sequencing and bioinformatic analysis

Blood samples were collected from the patient and his parents. Genomic DNA was extracted from whole blood using the QIAamp DNA Blood Mini Kit (Catalog number 51104; Qiagen). Three micrograms of genomic DNA was used for sequencing and the DNA library for exome sequencing was prepared using the Agilent Technologies SureSelect^XT^ Human All Exon V5 kit. Paired-end sequencing was performed on the Illumina HiSeq 2500 (Illumina) with read length of 100 bp for the whole exome using the TruSeq Cluster Kit v3 (Catalog number PE-401-3001; Illumina, Inc.), following the manufacturer's protocols.

The analysis of sequencing data, variant calling, and variant filtering were carried out as described previously (Muthusamy et al., 2017). Sanger sequencing was performed to validate the *IL2RG* variant in the patient using the forward primer (5′-GGCTGCACTTCTGGACTTTA-3′) and reverse primer (5′-CTAGATTTCTTCCTGACCAC-3′).

## Results and Discussion

Whole-exome sequencing of the patient and both of his parents resulted in an average of 96 million paired-end reads. Approximately, 96% reads were high quality (q ≥ 20) and aligned to the reference genome (hg19). A mean depth of 95 × was obtained across the three samples. The genotyped variants of the patient, mother, and father were subjected to joint variant calling using Genome Analysis Toolkit. We obtained 108,482 variants, of which 17,688 variants were located in the X-chromosome. We used ANNOVAR to annotate the variants and identified 60,354 exonic, 272 splice sites, and 519,518 intronic variants across samples. Furthermore, common variants with minor allele frequency >0.01 were removed after comparing the variants with common variants' databases such as 1000 Genome Project (Abecasis et al., 2012), NHLBI-EVS Exome Sequencing Project (http://evs.gs.washington.edu/EVS), and ExAC database (Lek et al., 2016).

Filtering of all synonymous variants resulted in 1341 variants, of which 1302 were exonic and 39 were splice-site variants. Based on the pedigree and symptoms exhibited by the proband, we investigated X-linked recessive as well as the autosomal recessive modes of inheritance. We obtained nine X-linked recessive and three autosomal recessive variants (Table 2). These variants were further shortlisted based on the known immunologic functions of the genes.

**Table 2.** List of Shortlisted Variants After Segregation Analysis

*Gene symbol*	*Locus*	*Nucleotide change*	*Protein change*	*Mutation type*	*Inheritance*	*Predicted effect of mutations*
*RNF19B*	chr1	g.33430102T>G	p.Gln62Pro	Nonsynonymous SNV	Autosomal recessive	Nonpathogenic
*ADGB*	chr6	g.147073796G>T	p.Ala1166Ser	Nonsynonymous SNV	Autosomal recessive	Deleterious
*FGF3*	chr11	g.69625252G>A	p.Arg181Cys	Nonsynonymous SNV	Autosomal recessive	Deleterious
*CSPG4*	chr15	g.75974771G>A	p.Leu1605Phe	Nonsynonymous SNV	Autosomal recessive	Deleterious
*MPRIP*	chr17	g.17039562delCAG	p.178_179del	Nonframeshift deletion	Autosomal recessive	Nonpathogenic
*FANCB*	chrX	g.14868681C>T	p.Arg481His	Nonsynonymous SNV	X-linked recessive	Nonpathogenic
*PRRG1*	chrX	g.37312617C>G	p.Pro134Ala	Nonsynonymous SNV	X-linked recessive	Nonpathogenic
*USP9X*	chrX	g.41022065A>C	p.Gln640His	Nonsynonymous SNV	X-linked recessive	Nonpathogenic
*CHST7*	chrX	g.46433596G>T	p.Gly77Val	Nonsynonymous SNV	X-linked recessive	Nonpathogenic
*VSIG4*	chrX	g.65247333T>A	p.Thr171Ser	Nonsynonymous SNV	X-linked recessive	Nonpathogenic
*IL2RG*	chrX	g.70330764G>T	p.Asn84Lys	Nonsynonymous SNV	X-linked recessive	Deleterious
*ATP7A*	chrX	g.77289224A>G	p.Asp1139Gly	Nonsynonymous SNV	X-linked recessive	Nonpathogenic
*ESX1*	chrX	g.103495266_103495267insCCGGGTGGCACAGGCGCCATGCGTGAG	p.G288delinsGSRMAPVPPG	Nonframeshift insertion	X-linked recessive	Nonpathogenic
*MAP7D3*	chrX	g.135310894C>T	p.Gly592Ser	Nonsynonymous SNV	X-linked recessive	Deleterious

*IL2RG*, interleukin 2 receptor subunit gamma.

**FIG. 2.**
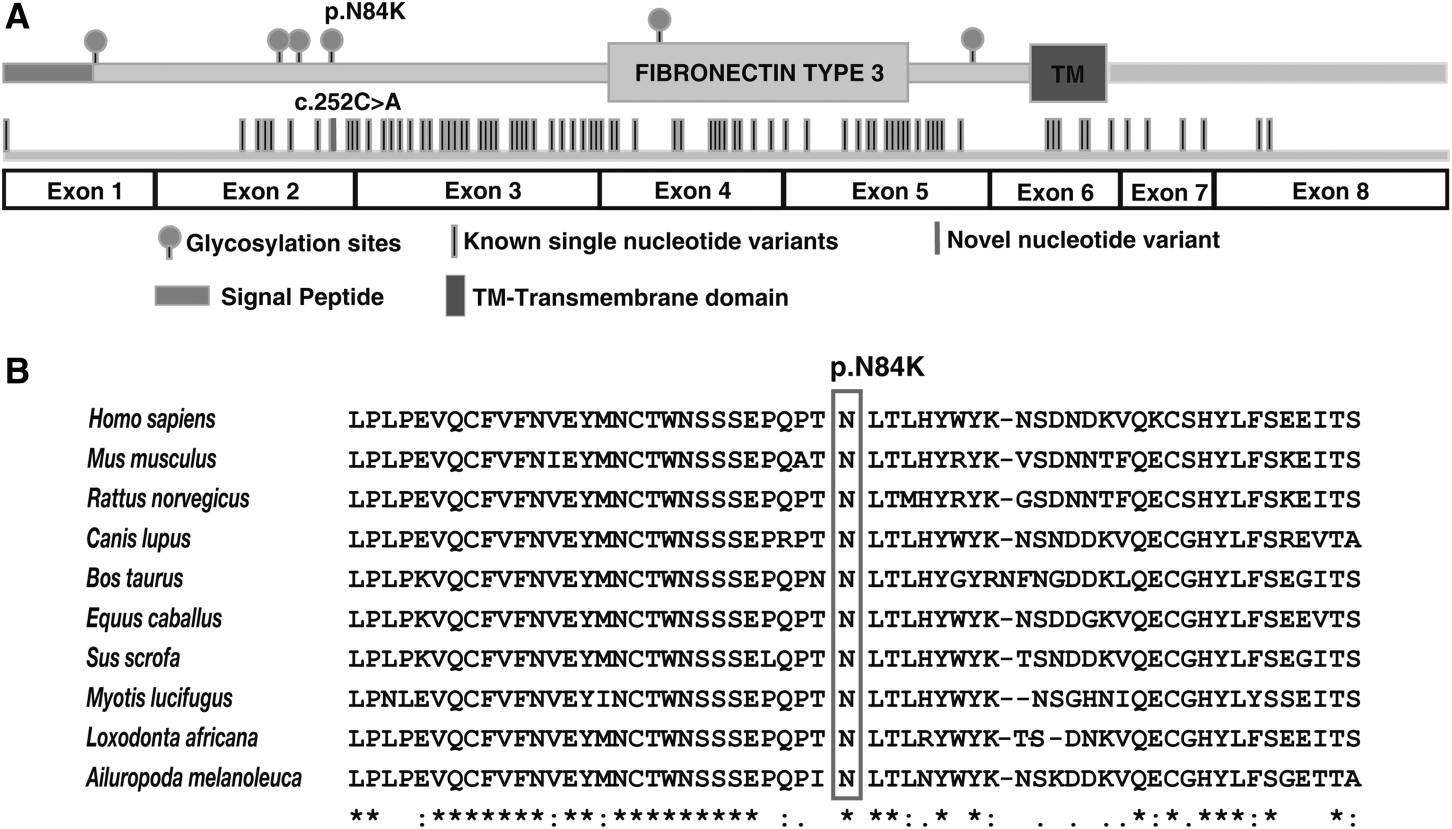
Depiction of *IL2RG* gene mutation in the context of protein architecture and sequence conservation. **(A)** A schematic representation of the IL2RG protein. The *top part* shows the domain architecture that includes signal peptide (*red*), fibronectin type 3 domain, and transmembrane domain (TM). The location of glycosylation sites is also indicated (ball and stick representation). The *middle part* shows the novel missense mutation identified in this study along with previously reported missense mutations. The *lower part* shows the spliced exonic architecture of the *IL2RG* gene. **(B)** Depiction of conservation of the asparagine residue, which was found to be mutated to lysine in the affected infant, across species. The conserved asparagine is indicated by a *rectangular box*.

We noted a potential causal variant, g.70330764G>T, in exon 2 of the *IL2RG* gene located at Xq13.1 (Fig. 1B). This mutation replaces cytosine with adenine at position 252 (RefSeq Accession: NM_000206.2), resulting in an amino acid substitution of asparagine (neutral, hydrophilic) to lysine (positive, hydrophilic) at position 84 (p.Asn84Lys). The variant was hemizygous in the affected individual, heterozygous in the mother, and was not found in the father, confirming X-linked recessive mode of inheritance. Sanger sequencing confirmed the nucleotide substitution (Fig. 1C). The missense variant p.Asn84Lys is found at a predicted glycosylation site (UniProt ID: P31785) located in the conserved extracellular domain (23–262) (Fig. 2A).

A multiple sequence alignment of the IL2RG protein with other species showed conservation of the asparagine residue, which was mutated in the patient (Fig. 2B). SIFT (Sorts Intolerant From Tolerant), PolyPhen-2, and MutationTaster concurred in their prediction the effect of the mutation as potentially pathogenic (Table 3). This variant was not found in the common variant databases, including dbSNP, 1000 Genomes Project, ExAC, or EVS. Together, these results indicate that the mutation has occurred in a potentially functional amino acid and may lead to a deleterious effect on the protein function.

**Table 3.** Prediction of Functional Effect of p.Asn84Lys Mutation in Interleukin 2 Receptor Subunit Gamma

*Gene symbol*	*Accession*	*Nucleotide change*	*Amino acid change*	*SIFT*	*PolyPhen-2*	*MutationTaster*	*Common variants: Minor allele frequency*
*IL2RG*	NM_000206	c.252C>A	p.Asn84Lys	Deleterious	Damaging	Disease causing	0

SIFT, Sorts Intolerant From Tolerant.

In summary, we identified a novel nucleotide variant c.252C>A (NM_000206.2), which results in a codon change from AAC to AAA leading to an amino acid substitution of asparagine to lysine (p.Asn84Lys). Fugmann et al. (1998) used nonradioactive single-strand confirmation polymorphism followed by sequencing and found a different nucleotide change (c.266C>G; NM_000206.1), corresponding to a codon change from AAC to AAG resulting in the same amino acid substitution that we have observed. Thus, our report describes a novel nucleotide variant in the *IL2RG* gene. Knowledge of this novel mutation is relevant for future targeted diagnostics that might employ the use of specific primers to amplify known nucleotide variants using polymerase chain reaction (PCR)-based techniques.

This report is also the first familial study showing the segregation of this mutation in the patient and his parents. This study reports the first mutation in *IL2RG* gene in an SCID patient from an Indian family. Because this is a rare disorder, it is important to note that our study serves also as a validation for the previous study that described p.Asn84Lys as a change in an SCID patient, indicating the causative nature of this mutation in SCID. Our study confirms that this mutation can be considered a causative mutation for SCID and used in clinical diagnosis of this condition as well for genetic counseling as the basis for initiating therapy.

## Conclusions

PID diseases are clinically and genetically heterogeneous for which accurate diagnosis is essential for improving therapeutic outcomes. SCID is potentially fatal, with mutations in *IL2RG* gene being the most common cause. Next-generation sequencing-based clinical diagnosis can greatly increase the diagnostic yield because multiple genes are simultaneously tested.

In this study, we applied next-generation sequencing on the patient and his parents and identified a novel nucleotide change (c.252C>A) that resulted in an amino acid change of asparagine to lysine at position 84. This amino acid substitution in the extracellular domain likely leads to an abnormal or unstable common gamma chain, thereby affecting signaling downstream of several interleukins (Conley et al., 1999). This novel variant can now be included in targeted sequencing-based assays in the setting of PIDs. Our report expands our understanding of currently known mutations in PID and underscores the power of next-generation sequencing as a potential diagnostic tool for PIDs.

## Abbreviations Used

ADA: adenine deaminase
AK2: adenylate kinase 2
CORO1A: coronin 1A
DCLRE1C: DNA crosslink repair 1C
IgA: immunoglobulin A
IgD: immunoglobulin D
IgE: immunoglobulin E
IgG: immunoglobulin G
IgM: immunoglobulin M
IL2RG: interleukin 2, receptor gamma
IUIS: International Union of Immunological Societies
JAK1: Janus kinase 1
JAK3: Janus kinase 3
LIG4: DNA ligase 4
MAPK: mitogen-activated protein kinase
NK: natural killer
PI3K: phosphoinositide 3-kinase
PIDs: primary immunodeficiencies
PRKDC: protein kinase, DNA-activated, catalytic polypeptide
PTPRC: protein tyrosine phosphatase, receptor type C
RAG1: recombination activating 1
RAG2: recombination activating 2
RBC: red blood cells
SCID: severe combined immunodeficiency
SIFT: sorts intolerant from tolerant
STAT5: signal transducer and activator of transcription 5
